# The neuroprotection of cerebrolysin after spontaneous intracerebral hemorrhage through regulates necroptosis via Akt/ GSK3β signaling pathway

**DOI:** 10.1590/ACB361002

**Published:** 2021-11-22

**Authors:** Yunna Tao, Yeping Xu, Meng Shen, Xiaoyan Feng, Yan Wu, Youping Wu, Liuyan Shen, Yuhai Wang

**Affiliations:** 1 BS. Department of Neurosurgery - Wuxi Clinical College of Anhui Medical University - 904th Hospital of Joint Logistic Support Force of PLA - Wuxi Clinical College of Anhui Medical University – Wuxi, China.; 2 BS. Department of Neurosurgery - Wuxi Clinical College of Anhui Medical University - 904th Hospital of Joint Logistic Support Force of PLA - Wuxi Clinical College of Anhui Medical University – Wuxi, China.; 3 BS. Department of Neurosurgery - Wuxi Clinical College of Anhui Medical University - 904th Hospital of Joint Logistic Support Force of PLA - Wuxi Clinical College of Anhui Medical University – Wuxi, China.; 4 BS. Department of Neurosurgery - Wuxi Clinical College of Anhui Medical University - 904th Hospital of Joint Logistic Support Force of PLA - Wuxi Clinical College of Anhui Medical University – Wuxi, China.; 5 BS. Department of Neurosurgery - Wuxi Clinical College of Anhui Medical University - 904th Hospital of Joint Logistic Support Force of PLA - Wuxi Clinical College of Anhui Medical University – Wuxi, China.; 6 BS. Department of Neurosurgery - Wuxi Clinical College of Anhui Medical University - 904th Hospital of Joint Logistic Support Force of PLA - Wuxi Clinical College of Anhui Medical University – Wuxi, China.; 7 BS. Department of Neurosurgery - Wuxi Clinical College of Anhui Medical University - 904th Hospital of Joint Logistic Support Force of PLA - Wuxi Clinical College of Anhui Medical University – Wuxi, China.; 8 BS. Department of Neurosurgery - Wuxi Clinical College of Anhui Medical University - 904th Hospital of Joint Logistic Support Force of PLA - Wuxi Clinical College of Anhui Medical University – Wuxi, China.

**Keywords:** Cerebral Hemorrhage, Brain Injuries, Necroptosis, Glycogen Synthase Kinase 3 beta, Mice

## Abstract

**Purpose:**

Spontaneous intracerebral hemorrhage (ICH) is a major cause of death and disability with a huge economic burden worldwide. Cerebrolysin (CBL) has been previously used as a nootropic drug. Necroptosis is a programmed cell death mechanism that plays a vital role in neuronal cell death after ICH. However, the precise role of necroptosis in CBL neuroprotection following ICH has not been confirmed.

**Methods:**

In the present study, we aimed to investigate the neuroprotective effects and potential molecular mechanisms of CBL in ICH-induced early brain injury (EBI) by regulating neural necroptosis in the C57BL/6 mice model. Mortality, neurological score, brain water content, and neuronal death were evaluated by terminal deoxynucleotidyl transferase dUTP nick end labeling (TUNEL) staining, Evans blue extravasation, Western blotting, and quantitative real-time polymerase chain reaction (PCR).

**Results:**

The results show that CBL treatment markedly increased the survival rate, neurological score, and neuron survival, and downregulated the protein expression of RIP1 and RIP3, which indicated that CBL-mediated inhibition of necroptosis, and ameliorated neuronal death after ICH. The neuroprotective capacity of CBL is partly dependent on the Akt/GSK3β signaling pathway.

**Conclusions:**

CBL improves neurological outcomes in mice and reduces neuronal death by protecting against neural necroptosis.

## Introduction

Spontaneous intracerebral hemorrhage (ICH) has the highest mortality rate among stroke subtypes, accounts for 15 to 20% of all stroke types, and has an increased incidence in elderly patients^1^-^4^. Acute ICH due to a large intracranial hematoma is associated with high morbidity and mortality, as it can lead to primary brain injury through the destruction of brain tissue and the high intracranial pressure (ICP) that results from the large hematoma^5^,^6^.

Previous studies revealed that craniotomy for hematoma evacuation is an effective therapy for limiting primary brain damage and decreasing ICP after ICH, which is of substantial interest^5^,^7^,^8^. However, craniotomy for hematoma evacuation cannot improve long-term outcomes and neurological recovery^9^. Increasing evidence shows that red blood cell debris, hemoglobin, its degradation products, and blood components trigger secondary brain injury following ICH and contributes to a series of damaging events, including neuroinflammation, brain edema, oxidative stress, blood-brain barrier (BBB) damage, and neuron death^10^-^15^. In recent years, an increasing number of studies have been conducted focusing on the mechanisms underlying ICH-induced secondary injury to search for better therapeutic targets for ICH. The possible mechanisms underlying early brain injury (EBI) include autophagy, apoptosis, direct neuronal death, and necroptosis^11^,^16^,^17^.

Cerebrolysin (CBL) is a small molecule peptide extracted from the porcine brain and has been previously used as a nootropic drug^18^. Increasing studies have demonstrated that CBL administration can promote recovery of motor function, improve EBI, decrease hippocampal neuronal death in basic researches^19^-^21^. DeBoer^19^ reported that poststroke CBL administration leads to recovery of motor function regardless rehabilitative training without a protective effect on stroke volume in a stroke model. In the seizure model, Kang^18^ also confirmed that CBL can decrease hippocampal neuronal death after the seizure. In recent clinical studies, CBL can improve overall outcomes after moderate to severe traumatic brain injury patients^22^,^23^ and was also safe, better for early rehabilitation patients after ischemic stroke^24^. Although the neuroprotection of effects, CBL treatment on the ICH is controversial. The neuroprotective mechanisms of CBL are also unclear.

Necroptosis is a newly discovered pathway of regulated necrosis, a caspase-independent programmed cell death mechanism that requires the proteins RIPK3 and MLKL and is induced by death receptors^25^. Increasing evidence suggests that necroptosis plays a critical role in central nervous system diseases, including traumatic brain injury^26^-^28^, ICH^29^,^30^, ischemic stroke^31^, amyotrophic lateral sclerosis, and Parkinson’s and Alzheimer’s disease^32^. The most upstream signaling activity required for the induction of necroptosis is the activation of a tumor necrosis factor (TNF) ligand family member (e.g., protein kinase function of receptor-interacting protein kinase-1–RIPK1 and mixed lineage kinase domain-like–MLKL). RIPK1 activation leads to necroptosis through the formation of a RIPK1–RIPK3 complex^33^. Necroptosis is common in early brain injury and may be an effective mechanism of ICH.

In this study, we investigated the neuroprotective effect of CBL therapy in a mice model of ICH, and whether the neuroprotection was dependent on the necroptosis pathway.

## Methods

The study protocol was approved by the Anhui Medical University-Affiliated Wuxi Clinical College Clinical Research Ethics Committee (YXLL-2020-016).

All animal experiments performed for this study complied with the National Institutes of Health guidelines for the handling of laboratory animals and were approved by the Ethics Committee of the Wuxi Medical College of Anhui Medical University. All experiments were conducted on healthy adult male C57BL/6J mice (22-25 g) (Anhui Medical University, Hefei, China). The mice were housed in animal care facilities with a 12-h light/dark cycle and had free access to food and water.

### ICH animal model

The ICH mouse model was generated based on a previously described protocol involving autologous blood injection^34^. Briefly, male C57BL6/J mice were anesthetized by intraperitoneal (i.p.) injection of 50 mg/kg pentobarbital sodium and placed in a prone position with a stereotactic head frame. The rectal temperature was kept at 37 ± 0.5°C during the operation using a heating pad. An artificial tear ointment was used to protect the eye from injury during surgery.

A midline scalp incision was made, and a cranial burr hole with a 1-mm diameter was made at the following coordinates relative to bregma: 0.2 mm posterior, 2.2 mm lateral to bregma, and 3.5 mm below the dura. A total of 30 μL of autologous blood without anticoagulation was collected from the caudal artery and rapidly injected into the basal ganglia through the burr hole via the 26-gauge needle of a 10-μL Hamilton syringe.

First, 5 μL of arterial blood was injected at a depth of 2.8 mm from the dura (injection speed: 3 μL /min). Five minutes later, the other 25 μL of blood was injected at a depth of 3.5 mm (injection speed: 3 μL /min). After the injection of autologous blood, the needle was kept in the brain for 10 min to prevent blood backflow along the needle tract. Finally, the hole was covered with medical bone wax. The animals in the Sham group received similar surgical procedures, but were injected at the same site with an equal volume of sterile saline instead of blood.

### Drug preparation and administration

**Figure 1:**
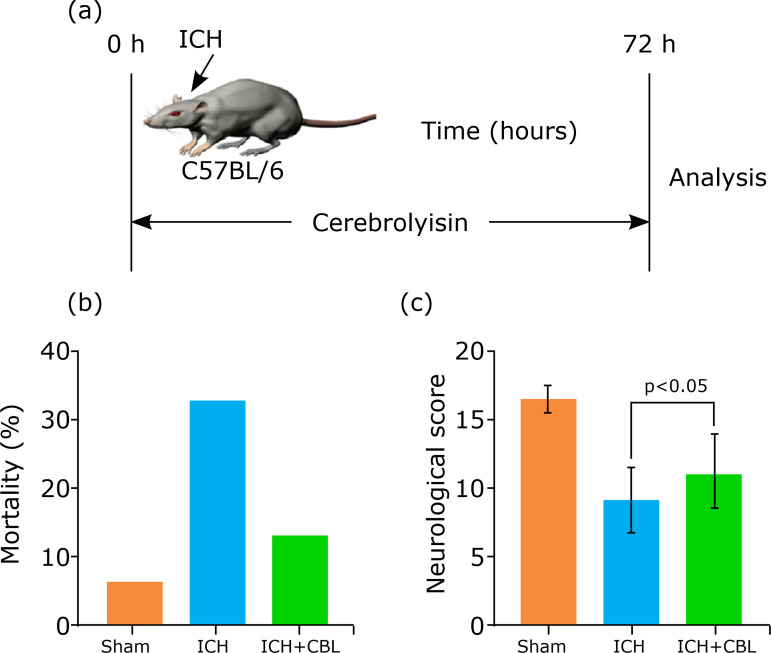
The effects of mortality and neurological function in the ICH models. **(a)** Schematic of experimental paradigm for modeling ICH in mice. **(b)** The mortality rates were increased in the SAH group, and decreased after CBL treatment, but no significant difference compared with the ICH group. **(c)** Neurological score of mice in Sham group, ICH group, and ICH+CBL group at 72 h after ICH (n=10, p <0.05). ANOVA; mean ± SEM.

ICH: spontaneous intracerebral hemorrhage; CBL: cerebrolysin; ANOVA: analysis of variance; SEM: scanning electron microscope.

After the ICH model was established successfully, animals were given daily intraperitoneal injections of either CBL (2.5 mL/kg/day, no dilution; EBEWE Arzneimittel, Austria) or plain (control) saline for 72 hours. Figure 1a summarizes the timeline of the experimental protocol of the study. We administered Ly294002 (2-(4-morpholinyl)-8-phenyl-4H-1-benzopyran-4-one) (Cell Signaling Technology, United States of America), which is a highly selective inhibitor of PI3K. Anesthetized mice were positioned in a stereotaxic frame, and Ly294002 (50 mmol in 25% dimethyl sulfoxide and phosphate-buffered saline–PBS) was injected intracerebroventricularly (10 mL, bregma; 1.4 mm lateral, 0.8 mm posterior, 3.6 mm deep) with a syringe pump 30 minutes before ICH.

### Neurobehavioral assessment

The severity of brain injury was evaluated by determining neurological function 72 hours after ICH using our previously described neurological grading system. The neurological scores ranged from 3 to 18 and included spontaneous activities (0-3), movement symmetry of all limbs (0-3), forelimb outstretching (0-3), body proprioception (1-3), response to vibrissae touch (1-3), and climbing (1-3). All mice from every group received a behavioral assessment, and a higher score represented better neurological function.

### Brain water-content measurement

The severity of brain edema was evaluated by measuring the brain water content using the standard wet-dry method, as previously reported^16^,^35^,^36^. The mice were sacrificed 72 hours after ICH, and the entire brain was harvested and separated into the ipsilateral and contralateral cortex, ipsilateral and contralateral basal ganglia, and cerebellum (wet weight). Then, brain specimens from each group were dehydrated at 105°C for 24 h to acquire the dry weight. The percentage of brain water content was equal to (wet weight – dry weight)/wet weight × 100%.

### Evans blue extravasation

Evans blue extravasation was performed as previously described^37^. Briefly, mice were anesthetized by pentobarbital sodium (50 mg/kg) injection 72 h after ICH. Evans blue dye (2%, 5 mL/kg; Sigma-Aldrich, St. Louis, MO, United States of America) was injected into the left femoral vein over 2 min and circulated for 60 min. Then, the mice were sacrificed with 100 mg/kg sodium pentobarbital via intraperitoneal injection and with PBS intracardial perfusion. Death was clarified by observing respiration and by using the corneal reflection method. The brains were removed and quickly divided into the left and right cerebral hemispheres, weighed, homogenized in saline, and centrifuged at 10,000 rpm for 30 min. Subsequently, the resultant supernatant was added with an equal volume of trichloroacetic acid, incubated overnight at 4°C, and centrifuged at 10,000 rpm for 30 min. Next, the resultant supernatant was collected and spectrophotometrically quantified at 610 nm for Evans blue dye.

### TUNEL staining

A terminal deoxynucleotidyl transferase dUTP nick end labeling (TUNEL) assay was conducted to assess neuronal death in the hippocampus. TUNEL reaction mixture (50 μL ) was added to each sample, and the slides were incubated in a humidified dark chamber for 60 min at 37°C. The slides were then incubated with 4′,6-diamidino-2-phenylindole (DAPI) for 5 minutes at room temperature in the dark to stain the nuclei, followed by imaging with a fluorescence microscope. The procedure was performed according to the manufacturer’s instructions with a TUNEL staining kit. A negative control (without the TUNEL reaction mixture) was used. The cell count was confirmed in four randomly selected high-power fields, and the data obtained from each field were averaged.

### Quantitative real-time PCR

Quantitative real-time polymerase chain reaction (qPCR) analysis was performed as indicated previously^38^. Total RNA was extracted from either cell cultures or hippocampal brain samples using TRIzol reagent (Gibco; Thermo Fisher Scientific, Inc., Waltham, MA, United States of America) according to the manufacturer’s instructions. Then, RNA was reverse transcribed to complementary DNA (cDNA) using the RevertAid First Strand cDNA Synthesis Kit (K1622; Thermo Fisher Scientific Inc., Rockford, IL, United States of America). ATF4 and CHOP mRNA levels in each sample were measured by qPCR using SYBR Green Master Mix (Toyobo Co., Ltd., Osaka, Japan). Glyceraldehyde 3-phosphate dehydrogenase (GAPDH) was used as an internal control. The qPCR thermocycling conditions were as follows: 45 (2 min) and 95°C (10 min), followed by 40 cycles of denaturation at 95°C (15 sec), annealing at 60°C (1 min), and extension at 72°C (1 min). All samples were analyzed in triplicate.

### Western blot analysis

Western blot analysis was performed as indicated previously^36^. Briefly, cerebral cortex samples or cell homogenates were collected, dissolved, and separated by sodium dodecyl sulfate-polyacrylamide gel electrophoresis in 10% polyacrylamide gels. A BCA Protein Assay Kit (Beyotime) was used to measure protein concentrations by the bicinchoninic acid method. Then, protein samples were transferred onto immobilon nitrocellulose membranes.

The membranes were blocked at room temperature for 1 h with 5% nonfat milk and then incubated with the following primary antibodies overnight at 4°C: rabbit anti-β-actin (1:1,000, rabbit polyclonal, Abcam, ab8227), rabbit anti-claudin-5 (1:1,000, rabbit monoclonal, Abcam, ab131259), rabbit ZO-1 (1:1,000, rabbit polyclonal, Abcam, ab 96587), rabbit anti-RIP1 (1:1,000; rabbit polyclonal; Abcam; cat. no. ab106393), rabbit anti-RIP3 (1:1,000; rabbit polyclonal; Abcam; cat. no. ab62344), rabbit anti-AKT (phospho S473) (1:1,000, rabbit monoclonal, Abcam, ab81283), and rabbit anti- Anti-GSK3β (phospho S9) (1:1,000, rabbit monoclonal, Abcam, ab75814).

After washing the membranes with tris-buffered saline with Tween (TBST) three times, HRP-conjugated goat anti-rabbit IgG or goat anti-mouse IgG secondary antibodies (1:5,000) were applied, and the membranes were incubated in the secondary antibodies at room temperature for 1.5 h. The protein bands were detected using a Bio-Rad imaging system (Bio-Rad, Hercules, CA, United States of America) and quantified with ImageJ.

### Statistical analysis

All experiments were repeated more than three times, and the data are expressed as the means and scanning electron microscope (SEM). Statistical Package for the Social Sciences (SPSS) 14.0 (SPSS, Chicago, IL, United States of America) and GraphPad Prism 6 (GraphPad Software, San Diego, CA, United States of America) were used for the statistical analyses. Student’s t-test was used if two groups were compared, and one-way analysis of variance (ANOVA) followed by Bonferroni’s post hoc test was used for the comparison of two independent variables. For nonnormally distributed data and/ornon-homogeneous variance, we used the Kruskal-Wallis test followed by Dunn’s post hoc test. For all the statistical analyses, data were considered significant at p < 0.05.

## Results

### The effects of mortality and neurological function in the ICH models

We constructed the ICH model and the CBL treatment after ICH (Fig. 1a). We evaluated the effect of CBL treatment on long-term neurological damage parameters, including mortality rates and neurological scores. As shown in Fig. 1, mortality rates (Fig. 1b) were decreased in the ICH+CBL group, but no significant difference compared with the ICH group (odds ratio–OR=0.308, 95% prediction interval–95%PI 0.049–1.928). Neurological scores were decreased significantly after ICH, and CBL treatment could significantly increase the neurological scores (p<0.05) (Fig. 1c).

### CBL alleviates brain edema and BBB permeability after ICH

**Figure 2:**
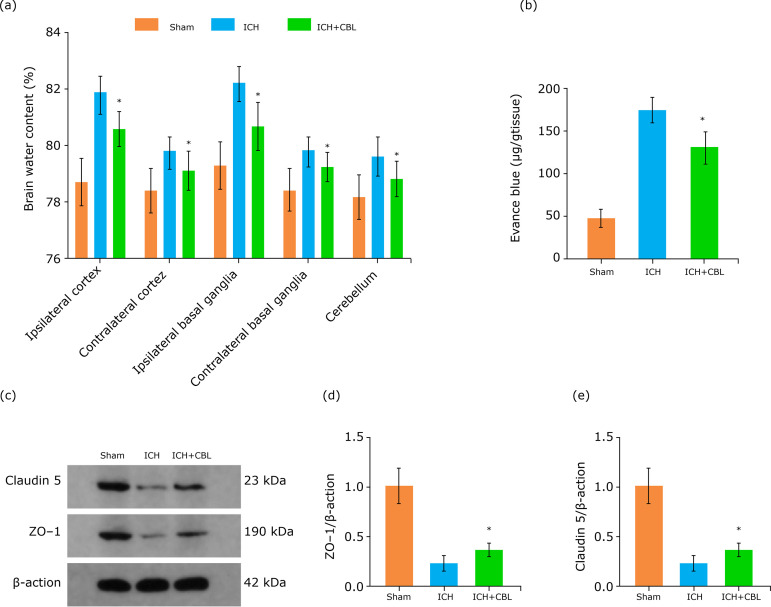
CBL alleviates brain edema and BBB permeability after ICH. **(a)** CBL alleviates brain water content after ICH. **(b)** CBL alleviates BBB permeability after ICH. **(c)** Expression of ZO-1 and Claudin 5 in the brain cortex of mice after ICH were determined by Western blotting. **(d-e)** Quantification of ZO-1 and Claudin 5 in the brain cortex to β-actin loading control, CBL increased ZO-1 and Claudin 5 expression after ICH in mice (n=5, *p <0.05 *vs*. ICH group; ANOVA; mean ± SEM).

ICH: spontaneous intracerebral hemorrhage; CBL: cerebrolysin; BBB: blood brain-barrier; ANOVA: analysis of variance; SEM: scanning electron microscope.

To clarify the EBI after ICH, we used brain water content by the wet-dry method at 72 h after ICH to evaluate brain damage. The results showed that ICH increased the brain water content significantly, which was alleviated after CBL treatment (Fig. 2a). Similar results in BBB permeability, which were increased significantly after ICH, and CBL administration could significantly alleviate BBB permeability (Fig. 2b).

To further clarify the BBB integrity, we detected the expression levels of Zonula occludens-1 (ZO-1) and Claudin 5 by Western blotting (Fig. 2c). The Western blotting results showed that the expression of tight junction proteins (Fig. 2 d-e) significantly decreased after ICH (p<0.05 *vs*. control group), suggesting that ICH-induced alterations in tight junction proteins may be responsible for the increased BBB permeability. Meanwhile, CBL treatment markedly alleviated this decrease at 72 h (*p<0.05 *vs*. ICH group).

### CBL alleviates hippocampus neuron necroptosis after ICH

Our previous study has indicated that necroptosis plays a vitally important role in early brain injury^27^. Therefore, the present study also investigated whether CBL improves brain injury after ICH via regulating neuron necroptosis.

**Figure 3:**
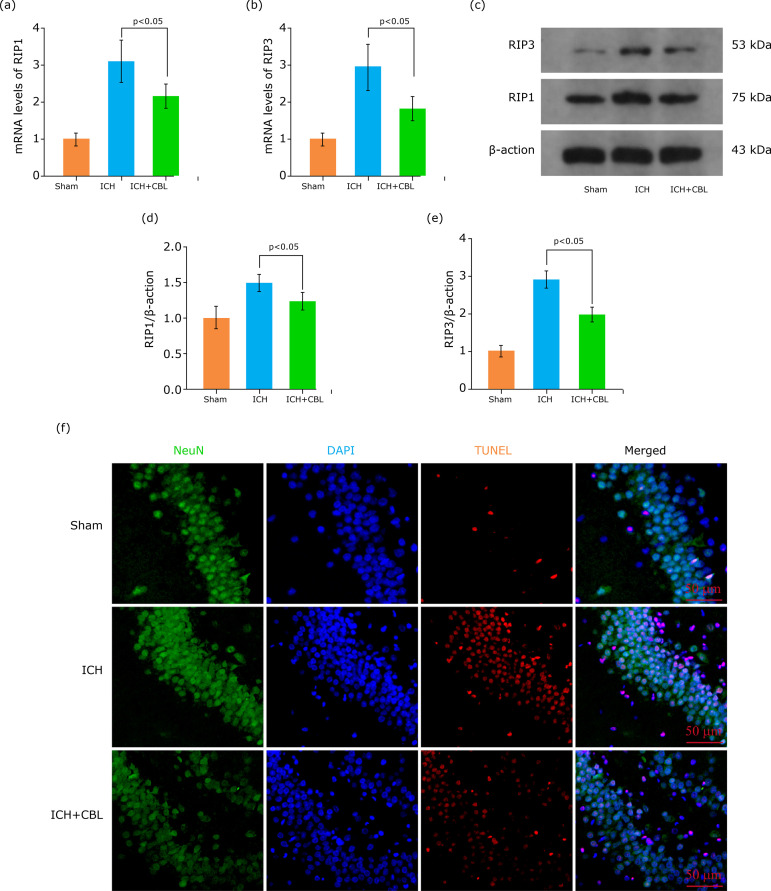
CBL alleviates hippocampus neuron necroptosis after ICH. **(a)** mRNA expression of RIP1 in the hippocampus of mice after ICH were determined by real-time PCR, CBL decreased the mRNA expression of RIP1 after ICH significantly. **(b)** CBL decreased the mRNA expression of RIP3 after ICH significantly. **(c)** Expression of RIP1 and RIP3 in the hippocampus of mice after ICH were determined by Western blotting. **(d-e)** Quantification of RIP1 and RIP3 in the hippocampus to β-actin loading control, CBL decreased hippocampus RIP1 and RIP3 expression after ICH significantly. **(f)** TUNEL staining, CBL alleviates neuronal death in the hippocampus 72 h after ICH. Representative images of apoptotic neurons are shown. p<0.05. Scale bar=50 μm. ANOVA; mean±SEM; DAPI; TUNEL.

ICH: spontaneous intracerebral hemorrhage; CBL: cerebrolysin; PCR: polymerase chain reaction; ANOVA: analysis of variance; SEM: scanning electron microscope; DAPI: 4′,6-diamidino-2-phenylindole; TUNEL: terminal deoxynucleotidyl transferase dUTP nick end labeling.

First, the mRNA expression levels of the necroptosis-related genes RIP1 and RIP3 in the hippocampus were determined by real time qPCR. As expected, the mRNA expression levels of RIP1 and RIP3 mRNA increased after ICH and decreased after CBL treatment (Fig. 3 a-b). The protein expression levels of RIP1 and RIP3 in the hippocampus were also measured by Western blotting analysis (Fig. 3c). The results were similar to the mRNA analysis results (Fig. 3 d-e). Importantly, to further clarify the hippocampus neuron death after ICH, we used TUNEL assay to quantify the level of cell death in treated and untreated ICH mice at 72 h after model construction. As we expected, our results indicated more hippocampus neuron death after ICH, while decreased significantly after CBL treatment (Fig. 3f). Hence, these data showed that CBL can alleviate hippocampus neuron necroptosis after ICH.

### CBL maybe regulates necroptosis by Akt/GSK3β signaling pathway after ICH

**Figure 4:**
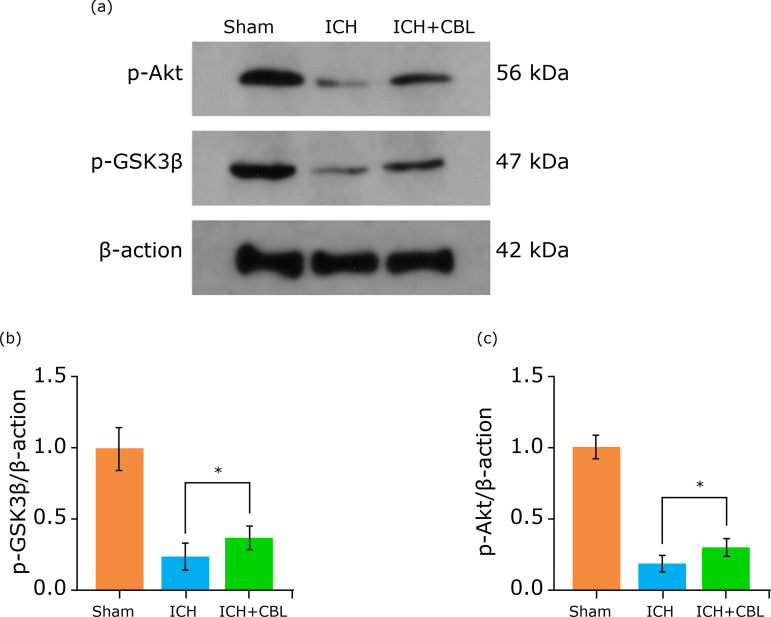
CBL maybe regulates necroptosis by Akt/GSK3β signaling pathway after ICH. **(a)** Expression of Akt and GSK3β in the brain cortex after ICH were determined by Western blotting. **(b-c)** Quantification of Akt and GSK3β in the brain cortex to β-actin loading control, CBL increased brain cortex Akt and GSK3β expression after ICH significantly. *p < 0.05; ANOVA; mean ± SEM.

CBL: cerebrolysin; ICH: spontaneous intracerebral hemorrhage; PCR: polymerase chain reaction; ANOVA: analysis of variance; SEM: scanning electron microscope.

Our previous study had demonstrated that necroptosis plays a key role in the pathogenesis of neuronal death after traumatic brain injury^26^. Then, we also detected the protein expression levels of Akt and GSK3β phosphorylation by Western blotting (Fig. 4a). The results showed that the expression levels of Akt and GSK3β phosphorylation were increased significantly in the ICH group and decreased after CBL administration (Fig. 4 b-c). Thus, these results showed that CBL may be inhibited SAH-induced necroptosis by regulated Akt/GSK3β signaling pathway.

### Ly294002 reversed the neuroprotection of CBL

**Figure 5:**
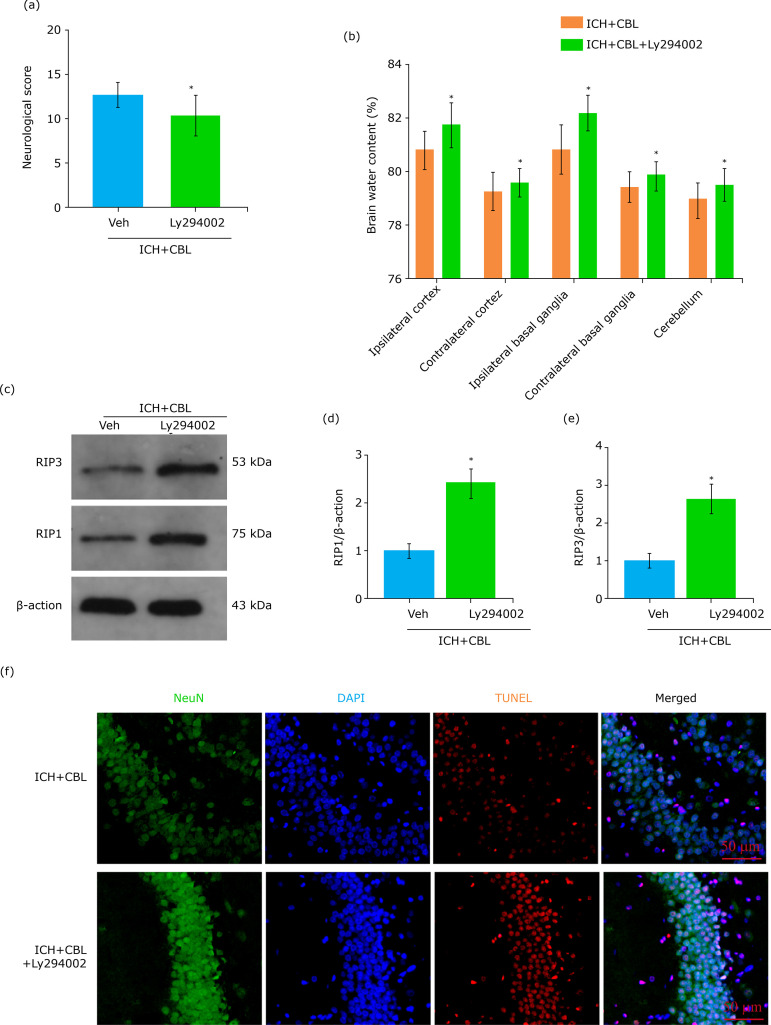
Ly294002 reversed the neuroprotection of CBL. **(a)** PI3K inhibitor, Ly294002 decreased the neurological score significantly at 72 h after ICH to compare with ICH group. **(b)** Ly294002 increased the brain water content significantly. **(c)** Expression of RIP1 and RIP3 in the hippocampus of mice after ICH were determined by Western blotting. **(d-e)** Quantification of RIP1 and RIP3 in the hippocampus to β-actin loading control, Ly294002 increased hippocampus RIP1 and RIP3 expression after ICH significantly. **(f)** TUNEL staining, Ly294002 increased neuronal death in the hippocampus 72 h after ICH, representative images of apoptotic neurons are shown. Scale bar = 50 μm; *p <0.05 *vs*. ICH+CBL group; ANOVA; mean ± SEM.

ICH: spontaneous intracerebral hemorrhage; CBL: cerebrolysin; DAPI: 4′,6-diamidino-2-phenylindole; TUNEL: terminal deoxynucleotidyl transferase dUTP nick end labeling; ANOVA: analysis of variance; SEM: scanning electron microscope.

To further explore the role of the Akt/GSK3β signaling pathway after CBL administration in the EBI after ICH. Ly294002, a highly specific PI3K inhibitor, can prevent phosphorylation of Akt and GSK3β. We infused Ly294002 into the left lateral ventricle of mice. The results showed that the behavioral scores and brain edema were improved after CBL treatment, while they were partially prevented by Ly294002 (Fig. 5 a-b). The expression levels of necroptosis-related protein (RIP1 and RIP3) also were decreased after Ly294002 treatment, and this effect was alleviated after Ly294002 (Fig. 5 c-e). We also performed TUNEL staining in brain sections (Fig. 5f), and the results showed that the anti-necroptosis role of CBL was partially inhibited by Ly294002.

## Discussion

Here, we evaluated the therapeutic potential of CBL for alleviating early brain injury in a mouse in the SAH model. The present study demonstrated that CBL was a neuroprotective agent that can attenuate early brain injury following SAH. We found that CBL can improve neurological dysfunction after SAH; CBL can alleviate brain edema and BBB permeability after SAH; CBL can prevent necroptosis and alleviate neuronal death after SAH; and the anti-necroptosis roles of CBL may be related to the Akt/GSK3β signaling pathway.

CBL is an intravenously administered small molecule peptide extracted from the porcine brain, composed of approximately 15% low molecular weight peptides and 85% amino acids in an aqueous solution and has been previously used as a nootropic drug^39^. It is a brain-specific pleiotropic agent that is proposed to target multiple pathways to improve functional recovery after neurological diseases and injuries in many central nervous system diseases^18^-^22^.

Satou *et al.* 40 reported that CBL can promote neurite outgrowth and cholinergic fiber regeneration in vitro. Guan *et al.* 41 also demonstrated that CBL can alleviate brain injury after focal cerebral ischemia by regulating neuroinflammation. The related molecular mechanisms partly via the activation of CREB/PGC-1α pathway play an important therapeutic role as anti-neuroinflammatory agents. In the spontaneously hypertensive rats with hyperglycemia model, CBL also can alleviate the reduction in the number of dendritic spines in the prefrontal cortex and hippocampus^42^.

In the clinical studies, Muresanu *et al.* 22 reported that CBL treatment is effective and safe for moderate to severe traumatic brain injury patients by a phase IIIb/IV single-center, prospective, randomized, double-blind, placebo-controlled clinical trial with 142 patients. An observational retrospective clinical study about patients also confirmed that CBL may improve the level of consciousness in stroke patients with a minimally conscious state^43^.

In the aneurysmal subarachnoid hemorrhage clinical retrospectively study, CBL injection during the acute period of SAH appeared to reduce the mortality rate, especially in poor-grade patients^44^, while a recent randomized, placebo-controlled, double-blind, pilot trial demonstrated that daily CBL (30 mL/day) is safe, well-tolerated, and feasible for SAH patients, but it does not improve the six-month global functional performance of patients^45^. In the present study, we found that CBL can improve early brain injury by alleviating brain edema and neuron necroptosis after SAH.

Necroptosis is a newly discovered pathway of regulated necrosis, a caspase-independent programmed cell death mechanism that requires the proteins RIPK3 and MLKL and is induced by death receptors^25^. Necroptotic cells display disrupted plasma membrane and cell lysis and can be observed in a variety of cell types, including neurons^27^. The most upstream signaling activity required a TNF ligand family member RIPK1 activation and led to necroptosis through the formation of a RIPK1–RIPK3 complex^33^.

Our previous study also demonstrated that necroptotic cell death might play an important role in traumatic neuronal injury-induced cell death and Si-Arc-induced aggravation of neuronal damage^27^.

Shen also reported that necroptosis is an important mechanism of cell death in brain injury after ICH, and inhibition of necroptosis may be a potential therapeutic intervention of ICH. Phosphorylation of RIP1 is the key molecular mechanism of necroptosis, which was activated in the in-vitro model of ICH^30^. Lu *et al.* 46 also indicated that microglial necroptosis is the main reason to lead brain injury in the pathogenesis of ICH. The potential mechanisms may be RIP3-mediated necroptosis by regulating the deubiquitinating enzyme A20 (also known as TNFAIP3) expression.

Our study also revealed that the protein and mRNA expression levels of RIPK1 and RIPK3 were elevated 72 h after the ICH procedure, and CBL decreased the expression of RIPK1 and RIPK3.

The regulated mechanisms of necroptosis are extremely complicated. In the present study, we found that the anti-necroptosis role of CBL may be related to the Akt/GSK3β signaling pathway. Chen *et al.* 26 reported that phosphorylation of Akt and GSK3β can regulate necroptosis after traumatic brain injury, and Akt inhibitor LY294002 partially reversed the protective effects of perampanel. Chen *et al.* 47 demonstrated that methylene blue can inhibit apoptosis and ameliorate neuroinflammation after ICH via activating the PI3K/Akt/GSK3β pathway. A recent study also indicated that Akt/p-GSKβ/β-catenin pathways play a very important role in the ICH. However, to date, there are no reports about the regulation of Akt/GSK3β on RIP1 activation, the exact mechanism of which needs to be further determined.

## Conclusions

Our study provided evidence that necroptosis, which is mediated by the RIP1 and RIP3, has been emerging as an important cellular regulatory mechanism and contributes to early brain injury after ICH. In this study, for the first time, we reported that CBL-induced regulation of necroptosis by Akt/GSK3β pathway and also provides a new idea to explore the biological effects and underlying anti-necroptosis and neuroprotection mechanisms of the CBL.
